# Prion disease: experimental models and reality

**DOI:** 10.1007/s00401-017-1670-5

**Published:** 2017-01-13

**Authors:** Sebastian Brandner, Zane Jaunmuktane

**Affiliations:** Department of Neurodegenerative Disease, UCL Institute of Neurology and Division of Neuropathology, The National Hospital for Neurology and Neurosurgery, University College London Hospitals NHS Foundation Trust, Queen Square, London, WC1N 3BG UK

## Abstract

The understanding of the pathogenesis and mechanisms of diseases requires a multidisciplinary approach, involving clinical observation, correlation to pathological processes, and modelling of disease mechanisms. It is an inherent challenge, and arguably impossible to generate model systems that can faithfully recapitulate all aspects of human disease. It is, therefore, important to be aware of the potentials and also the limitations of specific model systems. Model systems are usually designed to recapitulate only specific aspects of the disease, such as a pathological phenotype, a pathomechanism, or to test a hypothesis. Here, we evaluate and discuss model systems that were generated to understand clinical, pathological, genetic, biochemical, and epidemiological aspects of prion diseases. Whilst clinical research and studies on human tissue are an essential component of prion research, much of the understanding of the mechanisms governing transmission, replication, and toxicity comes from in vitro and in vivo studies. As with other neurodegenerative diseases caused by protein misfolding, the pathogenesis of prion disease is complex, full of conundra and contradictions. We will give here a historical overview of the use of models of prion disease, how they have evolved alongside the scientific questions, and how advancements in technologies have pushed the boundaries of our understanding of prion biology.

**Electronic supplementary material:**

The online version of this article (doi:10.1007/s00401-017-1670-5) contains supplementary material, which is available to authorized users.

## Glossary

Cellular prion protein (PrP^C^): Native, host-encoded, detergent soluble, protease-sensitive glycoprotein tethered to the cell membrane by a glycosylphosphatidylinositol (GPI) anchor. PrP^C^ is encoded by the *PRNP* gene in humans and the *Prnp* gene in mice. It has predominantly α-helical structure, and it is expressed at high levels in the brain.

Disease-associated prion protein (PrP^Sc^): Misfolded, post-translationally modified (abnormal) isoform of PrP^C^, with a substantial β-sheet content. It is insoluble in non-denaturating agents and shows partial protease resistance.

Prions: Operational term to denote the transmissible agent of prion disease. According to the protein-only hypothesis, an essential constituent of prions is PrP^Sc^.

Prion strains: Self-propagating prion protein conformations or assembly states. They are characterised by distinct neuropathological and biochemical profiles that can be maintained even after passage through an intermediate mammalian species with a different PrP amino-acid sequence.

Species barrier/transmission barrier: Barrier that limits cross-species infection, resulting in reduced transmission efficiency with lower attack rates and prolonged incubation periods.

Prion disease: Fatal neurodegenerative disorder, also known as transmissible spongiform encephalopathy, affecting humans and animals, characterised by accumulation of PrP^Sc^ in the brain. Human prion diseases develop sporadically can be inherited due to germ-line mutations in the *PRNP* gene and can be acquired through infection with human or animal prions through various routes.

Creutzfeldt–Jakob Disease (CJD): Human prion disease usually presenting as a rapidly progressive neurological disorder with hallmark features of dementia, ataxia, and myoclonus.

Sporadic CJD (sCJD): The most common type of human prion diseases of unknown aetiology, occurring worldwide with an annual incidence of 1–2 cases per million. The phenotypic and neuropathological heterogeneity observed in sCJD is thought to be related to different prion strains and genetic factors, in particular the polymorphism on codon 129 of the *PRNP* gene.

Sporadic Fatal Insomnia (sFI): Clinical syndrome and neuropathology similar to FFI found in patients with *PRNP* 129MM genotype, but with absence of a D178N or any other mutation in the *PRNP* gene. First described in 1999.

Variably Proteinase-Sensitive Prionopathy (VPSPr): Sporadic prion disease of unknown aetiology first reported in 2008 with distinctive biochemical properties of PrP^Sc^. In comparison with sCJD, PrP^Sc^ in VPSPr is less resistant to protease digestion. Neuropathology of VPSPr includes microplaques in thalamus and cerebellum.

Iatrogenic CJD (iCJD): Acquired prion disease caused by transmission of prions through medical procedures and treatments with prion-contaminated human cadaveric derived growth hormone, dura mater or cornea, or neurosurgical instruments.

Kuru: Prion disease acquired through dietary exposure to prions through ritualistic cannibalism practiced mainly by the people of the Fore linguistic group, a population of the Eastern Highlands of Papua New Guinea. Clinically, it is characterised by progressive cerebellar ataxia and less prominently, dementia. Kuru peaked in the 1950s before cannibalism ceased.

Variant CJD (vCJD): Acquired prion disease due to dietary exposure to BSE prions first recognised in 1996. It is characterised by involvement of the lymphoreticular tissue and by a distinct neuropathological picture often presenting in young people, usually with a characteristic clinical phenotype.

Inherited prion diseases (IPD): They are caused by germ-line mutations in the *PRNP* gene and are inherited in autosomal dominant fashion with a high penetrance. They present as distinct clinical syndromes (GSS, FFI, and CJD) depending on the mutation.

Gerstmann–Sträussler–Scheinker disease (GSS): Inherited prion disease characterised clinically by adult-onset ataxia, postural abnormalities, and cognitive decline. It is named after three clinicians, who first reported the disorder in 1936. A proline-to-leucine substitution at codon 102 (P102L) of human PrP is the most common mutation associated with the GSS phenotype and was first reported in 1989.

Fatal Familial Insomnia (FFI): Inherited prion disease characterised clinically by disruption of the physiological sleep patterns and is associated with a thalamic degeneration. It is caused by a *PRNP* D178N mutation on the allele harbouring methionine at polymorphic codon 129. FFI was first described in 1986 and was successfully transmitted to mice in 1995.

Bovine spongiform encephalopathy (BSE): Prion disease in cattle. BSE was epidemic in the United Kingdom with a peak incidence in 1992. More than 180,000 cattle were infected. Transmission of BSE prions through dietary exposure caused vCJD in humans.

Scrapie: Prion disease in sheep, known for more than two centuries. Scrapie was the first transmitted prion disease and scrapie prions and strains derived thereof have since been widely used in experimental studies.

## Introduction

Prion diseases are rare transmissible disorders, caused by misfolded post-translationally modified form of the native, host-encoded form of prion protein, designated PrP^C^ [2, 12]. It is proposed that the conversion of PrP^C^ into an abnormal isoform, commonly termed PrP^Sc^, is causally related to neurotoxicity, which is thought to be a result of a combination of toxic intermediates [157], overload of protein clearance pathways [106], and formation of protein aggregates [39].

The finding that the scrapie agent is infectious, transmissible, and resistant to proteinases, heat, and decontamination methods that modify nucleic acids and would normally kill any conventional microorganisms enabled Stanley Prusiner to formulate the protein-only hypothesis [146], previously proposed by Griffith [61] and to introduce the term “prion”—a small proteinaceous infectious particle devoid of nucleic acid. Subsequently, the *PRNP* gene and its product, PrP^C^, were identified [12, 36, 131]. It is now widely accepted that PrP^Sc^, the disease-associated isoform, aggregates and deposits in the brain, where it causes neurodegeneration. An enigmatic property of prions is the existence of strains which are associated with distinct neuropathological disease patterns and biochemical properties and are maintained through passages into different species, although often at a reduced efficiency, which has been termed species (or transmission) barrier [79]. Mutations in the open reading frame of the *PRNP* gene cause a spontaneous conversion of PrP^C^ into the abnormal isoform and often result in neurodegenerative syndromes, but are not always transmissible.

This review will give an overview of the historical development of models of prion disease, which has been generated to address the multiple aspects of prion disease. We will put the models in the context of the available technology and the scientific questions at the time. The use of model systems for a human disease usually aims at addressing specific biological questions, rather than being comprehensive or being a faithful replica of the human disease. We will describe here for which aspects these models are useful and where they fail to give an answer. Many models have been generated to address a specific hypothesis rather than replicating an existing disease, and some raise more questions than they answer. Whilst certain aspects can be addressed by cell culture or in cell-free systems in vitro, animal models remain a mainstay in prion disease research, as only these allow to study clinical phenotypes, neuropathological characteristics, transmission barriers, and the role of pathogenic mutations. In this review, we will first present the most significant animal models that have been used for the study of prion disease. Genetically modified mice have given answers to many essential biological questions in prion disease and will, therefore, form a major part of this review. Then, we will briefly discuss ex vivo and in vitro studies. Some of the biological questions can only be addressed by a combination of animal models and methodologies and these are discussed in the second part of the review. The illustrations of this review then combine and integrate the systematic overview of the model systems with the biological question.

In this review, we will give the reader an overview, and a selection of references will provide guidance for further reading. It is not our aim and also not possible to give an entirely exhaustive list of references, and we, therefore, had to resort to citing the most commonly used models or approaches. Where necessary, we have given references to in-depth reviews on specific topics.

## Animal models of prion disease: from monkeys to flies

### Transmission of human prion diseases into primates

**Fig. 1:**
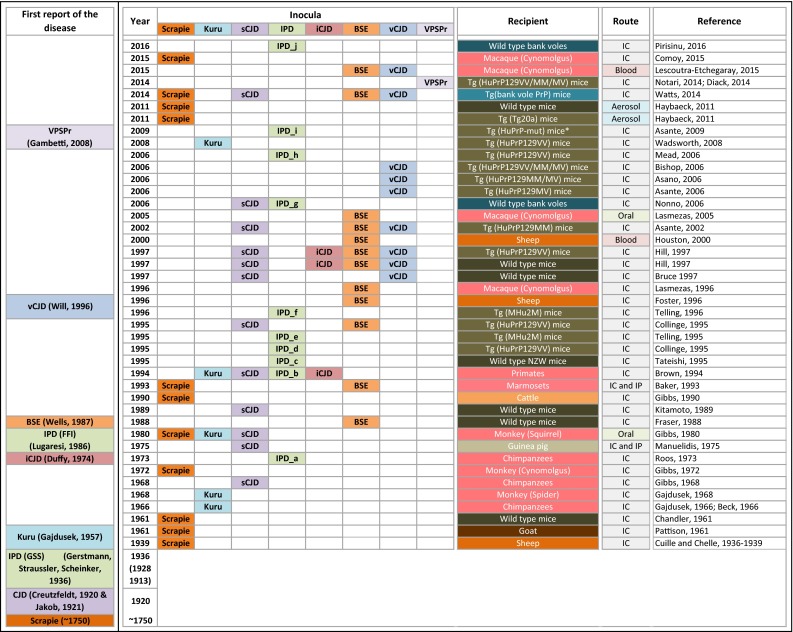
History of the use of animal models of prion disease. The *left column* indicates when the diseases were first reported, starting with scrapie in the 18th century and CJD in 1920. The time line (*second column*) is not strictly chronological, but is aligned with the generation of relevant animal models. The subsequent columns, titled “*inocula*”, indicate the different forms of prions that were transmitted. The column “*recipient*” indicates the species and, where appropriate, their genotypes. To the *right*, the transmission route and the reference. The inocula in the column IPD are complex and are according to the following key: *a* CJD in patients with family history (specific mutation not known); *b* E200K, D178N 129V, P102L, 5-OPRI, 7-OPRI, and 8-OPRI; *c* D178N 129M and 24 basepair deletion mutation on the same *PRNP* allele; *d* D178N 129M; *e* E200K; *f* D178N 129M; *g* E200K, V210I; *h* 6-OPRI; *i* P102L, A117V, and E200K; *j* P102L, A117V, and F198S; *Tg* (HuPrP-mut) mice recipients: *HuPrP-P102L, HuPrP-A117V, and HuPrP-E200K; abbreviations for routes of transmission: *IC* intracerebral, *IP* intraperitoneal

Inoculation of isolates of kuru and Creutzfeldt-Jakob disease (CJD) into primates [54, 55, 57] proved transmission of human prion diseases and provided a model for the reproduction of histological phenotypes of spongiform encephalopathy (Fig. 1). Even decades later, despite the growing importance of genetically modified mouse models, which provided increased versatility and better readout, primates continued to have a role in transmission studies: transmission of brain extracts from cattle with Bovine Spongiform Encephalopathy (BSE) into macaques established the first pathogenetic link between BSE and variant CJD (vCJD) [101], by producing a pathological phenotype that was nearly identical to vCJD. In more recent studies, primate models are used in correlative studies looking at transmission properties of different prion strains [135], to establish the zoonotic potential of transmissible spongiform encephalopathies (TSE) other than BSE, such as natural scrapie [46], to assess transfusion safety through application of specifically designed prion removal filters [104], or to estimate the risk of oral infection with the BSE agent [100]. Cynomolgus macaque monkey (*Macaca fascicularis*) has remained a relatively popular choice for prion research in primates, due to their longevity, phylogenetic proximity to humans, similar digestive physiology, and their genetic similarity to humans, including homozygosity for methionine at *PRNP* codon 129. The main limitations for more experimental studies on primates are their expensive maintenance, duration of experiments, and ethical concerns.

### Rodents (hamsters, mice, and bank voles): modelling transmission, disease mechanisms, and function of PrP

**Fig. 2:**
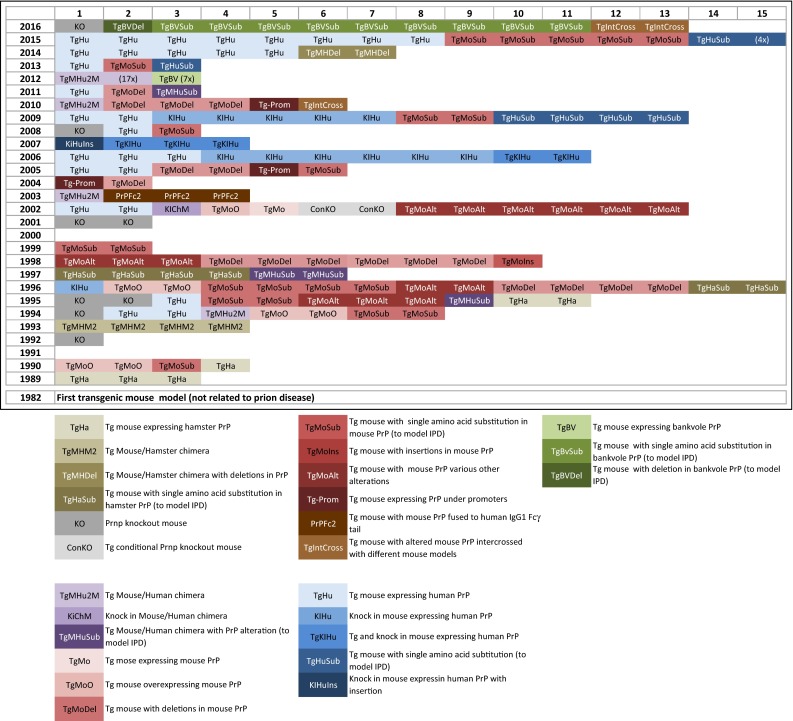
Detailed overview of genetically modified models in prion research. 1982 denotes the year of the first report of the generation of a transgenic mouse. The first use of transgenic mice in prion research was in 1989. The legend explains the abbreviation used in the main graph. Multiple fields indicate the generation of multiple mouse lines, sometimes published in a single article (see also supplementary table for further details)

Hamsters used to be popular for many types of prion studies since the 1970s, but were gradually replaced by mouse models, due to the widespread availability of genetically modified mice in the 1990s, initially by transgenesis, followed by the combination with the gene knock-out model and, more recently, the inducible expression and deletion of the *Prnp* gene. A niche model, the bank vole will also be briefly discussed here (Fig. 2).

Wild-type Syrian golden hamsters have been used in prion research for over four decades with the first transmission experiments in the 1970s. Hamsters have been invaluable for the understanding of anatomical and functional pathways of prion spread from the periphery and within the CNS [75, 80, 81]. Through these models, distinct properties of different prion strains have been identified and they helped understanding the species barriers [78]. Although hamsters are still occasionally used in prion research, for example, for investigating the distribution and spread of prions after peripheral challenge [35], the wide availability and versatility of transgenic mouse models have made hamster models largely obsolete.

**Fig. 3:**
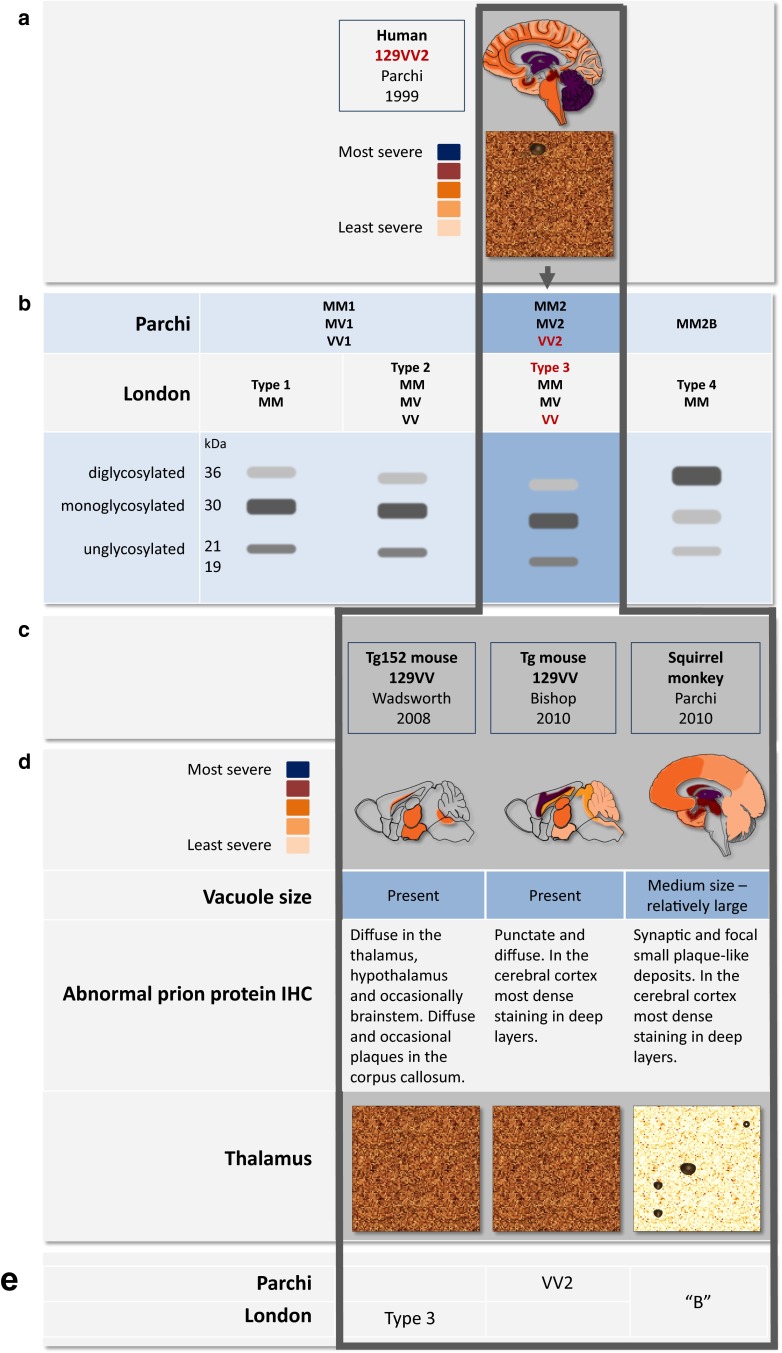
Comparison of neuropathology and molecular strain types between an sCJD patient with *PRNP* codon 129 VV genotype and animal models. **a** Schematic representation of the severity of prion pathology and characteristic immunohistological pattern of synaptic and plaque-like abnormal prion protein deposits in the brain from sCJD patient with *PRNP* codon 129VV genotype. **b** Corresponding molecular strain is VV2 (Parchi classification) or type 3 (London classification). **c** Three animal models, two humanised mouse lines with 129V genotype, and a squirrel monkey. **d** Schematic representation of the anatomical distribution of prion disease pathology, vacuolar size in the neuropil, and immunohistochemical pattern of abnormal prion protein deposits following disease development after intracerebral inoculation with the brain homogenate from the sCJD patient with 129VV genotype. **e** Corresponding molecular strain types in the animal models

Bank voles, small rodents, have been added relatively recently to the models of prion research [29, 128]. Their susceptibility to prions originating from multiple different species is thought to be encoded in the bank vole PrP at codon 109 [Methionine or Isoleucine (Ile)], which has been elegantly confirmed in mouse models expressing bank vole PrP (BVPrP) [188]. BVPrP with Ile at codon 109 causes a spontaneous neurodegenerative illness in mice, possibly due to an inherent propensity to misfold [189]. Furthermore, the primary transmission efficiency of sCJD prions (MM1, MV1, and MM2, but not MV2 and VV2 (Parchi classification [136], see also Fig. 3 for homologisation with the London classification) and the transmission of inherited prion disease (IPD) (V210I, E220K, A117V; P102L, and F198S) into bank voles is not dissimilar to that in humanised transgenic mice [128, 141]. Importantly, in bank voles, the neuropathological and biochemical strain properties are well preserved [128]. However, whilst these models show deposition of abundant abnormal prion protein in the brain [187], the neuropathological phenotypes and the biochemical characteristics of the modelled IPD do not correspond well to the human pathologies.

### Mouse models: a history of milestones in prion research

The initial use of mouse models was, as with other animals, for the transmission and adaptation studies by serial propagation of sheep scrapie to wild-type mice (Fig. 1; Table 1), to understand the biology of the species barrier and of prion strains. Whilst initially done on inbred mice, this has significantly changed with the availability of transgenic models (Fig. 2; Supplementary Table 1). The first transgenic mice in prion research, published in 1989—several years after the creation of the first transgenic animal in 1982 [134]—expressed hamster transgenes and were an important milestone towards the understanding of transmission barriers and strains [163], as they for the first time allowed to circumvent the species barrier and demonstrated the relevance of the amino-acid sequence of the *Prnp* gene for the susceptibility to prions strains. Expression of the murine equivalent of the human P102L mutation was the first significant attempt to model IPD associated with the Gerstmann–Sträussler–Scheinker (GSS) syndrome. Mice showed neurodegeneration, but no distinctive prion protein accumulation as seen in humans with GSS. GSS brain extracts could be propagated within the same strain of mice [69], but not into inbred wild-type CD1 mice [70]. This model expressed human *Prnp* transgenes on a background of murine *Prnp* sequences, and this caused a significant interference of the transgenic prion protein with its endogenous homologue (Table 1). A further milestone in prion research followed in 1992 with the generation of the *Prnp* knock-out (*Prnp* ^*0/0*^) mouse [28] (Fig. 2), which not only demonstrated the requirement of the normal host prion protein to propagate prions [155], but also set the foundation to a nearly limitless numbers of models expressing a multitude of transgenes without interference of the endogenous protein. Weissmann, the creator of the mouse has befittingly phrased it “a mouse to remember” [190]. Economical animal maintenance and the continuous advances in gene editing and manipulation techniques have made mice a widely accepted and preferred model system, not only in prion research, but nearly universally in life sciences. New mouse models are continuously generated to address the biology of strains, function of PrP, transmission barrier, and with attempt to recapitulate human disease. The following paragraphs will outline the role of wild-type inbred strains of mice, the role of knock-out, transgenic and gene targeted mice in modelling human prion disease, and the function of PrP.

**Table 1:** Most commonly used mouse models in prion research

Mouse models	Number of lines (approximate)	Advantages, main use	Disadvantages
Wild-type inbred mice	9	Consistent, controlled genetic background Litter mate controls for transgenic mice Scrapie model for testing anti-prion compounds	Resistance to infection with human prions (species barrier)
*Prnp* ^*0/0*^ mice + conditional post-natal *Prnp* knock-out mice	8 + 2	Study of physiological role of PrP^C^ and its role in PrP^Sc^ formation and disease; investigating potential therapeutic targets Practically all transgenic animals are bred into *Prnp* ^*0/0*^ background	Different targeting strategies have resulted in slightly different phenotypes and one major phenotype (Ngsk mice)
Humanised/human *Prnp* sequences expressed as transgene on the background of *Prnp* ^*0/0*^ mouse	45	Recapitulates neuropathological and biochemical features of human prion disease. Important for studying transmission properties, genetic susceptibility, strain types, including identification of new atypical strain types and unexpected phenotypes, investigation of zoonotic potential of, e.g., bovine, elk, sheep TSE, therapeutic compounds	Differences in genetic backgrounds; technological differences in constructed vectors; transgenic versus knock-in; different PrP expression levels These differences make it difficult to compare between different models
Mouse PrP overexpression on the background of *Prnp* ^*0/0*^ mouse	8	Investigating physiological role of PrP^C^, structure of PrP^C^ and PrP^Sc^, prion toxicity, potential therapeutic targets Screening of anti-prion drug compounds	Differences in genetic backgrounds; technological differences in constructed vectors; transgenic versus knock-in; different PrP expression levels Often do not recapitulate the neuropathology and biochemistry of human prion disease
Chimera: mouse/human PrP on the background of *Prnp* ^*0/0*^ mouse	22	More efficient propagation of human prions (in comparison with mouse PrP); shorter incubation periods, allowing to study infectivity (bioassays) and prion strains relatively rapidly; potential use for anti-prion drug compound screening	To a variable degree recapitulate neuropathological and biochemical aspects of human prion disease
Transgenic mouse with *Prnp* sequences from different species (syrian hamster, bovine, ovine, porcine, guinea pig, elk, bank vole), including chimeras (mouse/other species)	Numerous; The number of lines for each species is beyond the scope of this review	More efficient and rapid propagation of certain prions strains (e.g., vCJD propagate more efficiently in transgenic mice expressing bovine or guinea pig *Prnp* sequences)	Do not recapitulate precisely the neuropathology and biochemistry of human prion disease
Mouse PrP expressed under the control of tissue-specific promoters	3	Allows for the selective expression of functional or mutant PrP and allows for the detection of the role of PrP in propagation in the context of tissue compartments	Can be an artificial system which may not reflect the reality of prion biology
Mouse PrP with mutations (deletions, substitutions, insertions)	45	Many develop spontaneous disease; many develop distinct neuropathology. Served the understanding of function domains of PrP, and led to the discovery of spontaneous neurodegeneration caused by truncated PrP	Do not recapitulate any known pathology neuropathology or biochemistry of human prion disease; may lead to generation of conclusions which are irrelevant to human inherited prion disease
Chimera with mutations	2	Develop spontaneous disease; develop distinct neuropathology	Do not recapitulate particularly well the neuropathology and biochemistry of human prion disease
Humanised mice with mutations (deletions, substitutions, insertions)	10	Develop neuropathology which is most similar to human disease	Do not develop spontaneous disease
Bank vole PrP with mutations (deletions, substitutions, insertions)	3	Develop spontaneous disease; develop distinct neuropathology	Do not recapitulate particularly well the neuropathology and biochemistry of human prion disease; may lead to generation of conclusions which are irrelevant to human inherited prion disease

Left column indicates the type of model, left centre column indicates the approximate number of published mouse lines, left right centre column the advantages and the main use of these models, and the right column the disadvantages or drawbacks of the model

### Inbred strains of wild-type mice

The commonest inbred strains used in prion research are C57Bl/6L, C57Bl/6N, C57BL/10, FVB, and 129/Ola, and nowadays, less commonly used strains are RIII, VM, NZW, and CD1. Initially, wild-type mice were used to transmit sheep scrapie and to generate and adapt scrapie strains for further propagation (Fig. 1). For example, the strain ME7 was the result of the transmission of natural scrapie of Suffolk sheep directly into mice. In contrast, other commonly used strains 22L, RML, and 79A, all distinct from ME7, share a common origin in that they were all derived from the Moredun Institute’s sheep scrapie brain pool (reviewed in [168], and a detailed experimental characterisation is given in [97]). Likewise, many of these inbred mouse strains were also used, albeit with much less success, to transmit and propagate human prions of various aetiologies, i.e., sporadic, inherited, and acquired forms, including vCJD [26, 64, 83, 172]. The species barrier between humans and mice (also described as transmission barrier) results in low attack rates and markedly prolonged incubation periods. At the same time, the recognition of the existence of a species barrier was an important milestone as it eventually led to the generation of “humanised” transgenic mice and also to the transgenic mapping of the regions of the prion protein relevant for transmission barriers.

### PrP knock-out mice (PrP-null mouse, *Prnp*^*0/0*^): a breakthrough in prion research to understand the role of PrP^C^

**Fig. 4:**
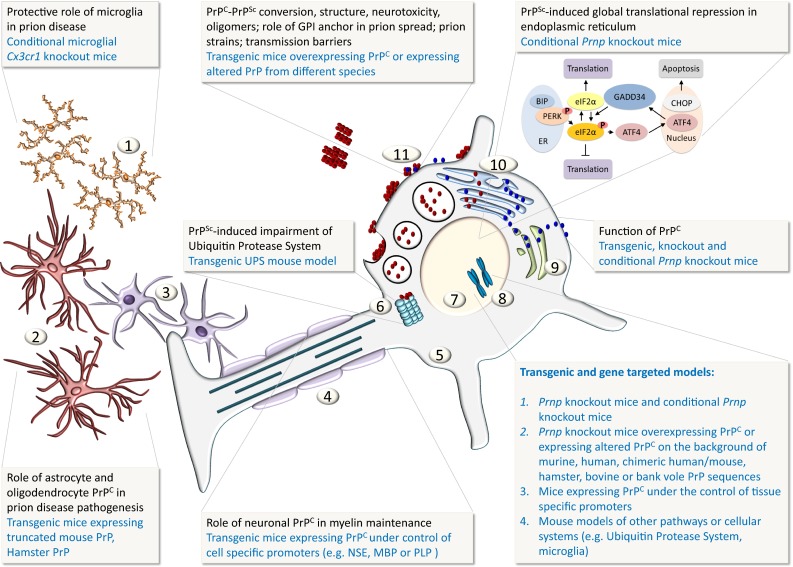
Schematic representation of the cellular and sub-cellular compartments and how PrP function and disease mechanisms are investigated with animal models. Key: *1* microglia, *2* astrocytes, *3* oligodendrocytes, *4* myelinated axon, *5* neuronal cytoplasm, *6* ubiquitin protease system (UPS), *7* neuronal nucleus, *8* chromosomes, *9* Golgi complex, *10* Endoplasmic reticulum (ER), *11 blue globules* represent native cellular prion protein PrP^C^, *red globules* represent misfolded prion protein PrP^Sc^

Since the publication of the first *Prnp* knock-out or *Prnp* null (*Prnp* ^*0/0*^) mouse in 1992 [27, 28, 155], seven additional *Prnp* ^*0/0*^ models have been reported as of 2016 (Fig. 2; Supplementary Table 1). All these models lack significant regions of the *Prnp* open reading frame and they have in common that they do not express PrP^C^. The two first published models were generated in Zürich (hence termed ZH or Zrch I *Prnp* ^*0/0)*^ and in Edinburgh (Edbg *Prnp* ^*0/0*^ [109]*)* and had only minor phenotypes [179] [Fig. 4(9)], whilst a model from Nagasaki, Japan (Ngsk *Prnp* ^*0/0*^) developed ataxia, caused by a progressive loss of cerebellar Purkinje cells [156]. It was later found that this was caused by the deletion of a splice acceptor in intron 2 of the *Prnp* gene, which causes an intergenic splicing event and overproduction of the transcript of a gene named *Doppel* or *Dpl* [124]. Expressed under normal circumstances at very low levels, *Dpl* is massively upregulated in the brain of Ngsk *Prnp* ^*0/0*^ mice due to the high activity of the *Prnp* promoter and results in the expression of Doppel protein in the CNS. A number of studies have then shown the role of Doppel protein in experimental neurodegeneration (for example, see [154]), and functionally intriguing links with truncated PrP^C^, which causes a similar cerebellar phenotype, have been made (see below), but a direct relevance to human neurological diseases could never be established [117]. Other confounding factors were the embryonic stem cells and breeding schemes used for the generation of *Prnp* ^*0/0*^ mice. The most popular embryonic stem cell lines are derived from the mouse 129Ola strain, whilst mouse lines are maintained in non-129 backgrounds. Using gene editing of C57BL/6J fertilised oocytes [130], a new *Prnp* ^*0/0*^ mouse (termed *Prnp* ^ZH3/ZH3^) has been recently generated, which has eliminated all phenotypes previously reported, except chronic demyelination of peripheral nerves. Thus, the authors conclude that the demyelinating neuropathy is indeed the (only) effect of the *Prnp* loss of function [Fig. 4(8, 9)].

### Human *PRNP* transgenesis on the background of *Prnp*^*0/0*^ mice: a leap forward to understand human prion disease

The generation of *Prnp* ^*0/0*^ mice was an essential tool to create transgenic mice lacking the endogenous murine PrP^C^. Transmission of human prions into wild-type mice was fraught with significant transmission barriers and long incubation times [174], and this obstacle was effectively removed by intercrossing transgenic mice expressing full-length human PrP or chimeric murine–human PrP (containing components of human and murine genome) with *Prnp* ^*0/0*^ mice (Fig. 1). These models had comparatively short incubation periods with a high attack rate, they developed clinical phenotypes, and were suitable for the study of transmission barriers from other species and from distinct human prion isolates, which they often faithfully replicated neuropathologically and biochemically. As a consequence, these models have a significant potential to test therapeutic compounds developed in silico and in vitro. In fact, these features render these models superior to those of many major neurodegenerative diseases, such as Alzheimer’s or Parkinson’s diseases, where clinical–pathological correlation between the animal model and human disease often so far remain less compelling.

At present, at least 45 humanised mouse lines have been generated with combined transgenic and gene targeting approaches on the basis of *Prnp* ^*0/0*^ mutants (Figs. 2, 3). The transgenic constructs not only carry the desired pathogenic mutations, but importantly the polymorphic genotypes at codon 129 (and more recently codon 219 and codon 127 G > V modification [9]) of the *PRNP* gene. Over the years, the importance of a well-controlled genetic background has been increasingly recognised. Early studies often used mixed backgrounds (e.g., the 129V line (Tg152) or the 129M line (Tg35) were initially kept in a FVB/N, C57BL, and 129Sv background [6]), whilst later, these lines were maintained on a strictly FVB-congenic background [9]. The comparability between different mouse lines is then further confounded by the different PrP expression levels. For example, Tg650 mice (HuM129, sixfold PrP expression) [16] inoculated with vCJD prions develop neurological disease with a 100% attack rate, but Tg35 mice (HuM129, two-fold PrP expression) [6] and knock-in mice (replacing murine with single copy human PrP) [18] only infrequently show neurological symptoms, whilst histological and biochemical examination clearly suggests subclinical disease. Likewise, transmission of sCJD, into the “knock-in” MM, MV, and VV models [19], does not result in clinical disease when inoculated with sCJD MM2 strains (Parchi classification [136], see also Fig. 3 for homologisation with the London classification). In contrast, Tg650 mice with much higher PrP levels develop clinical signs of prion disease with a mean incubation time of approximately 280 days [33]. Thus, for the purpose of disease modelling, for example, to assess drug effects on incubation time, PrP overexpression models have a clear advantage. This also is relevant for economical and practical reasons, such as cost of animal maintenance and the duration of typical research projects (e.g., life cycle of a grant or duration of a PhD). From a practical aspect, doubling the expression levels can often be relatively easily achieved by breeding mice into homozygous status. On the other hand, a model system that produces strictly physiological PrP expression levels, such as the knock-in model, does have a role for the study of function of PrP, but the limited lifespan of a mouse may preclude the development of a phenotype during lifetime.

### Chimeric human/mouse models on the background of* Prnp*^*0/0*^ mouse

Since the generation of the first transgenic mouse line expressing chimeric mouse and human PrP was reported in 1994 [174], over 20 different chimeric human–mouse lines, designated MHu2M (25% murine, 50% human, and 25% murine sequence) have been created. These lines differ in the amino-acid sequence of human PrP residues in the chimeric sequence, their PrP expression level, and incubation periods after inoculation with CJD prions [58]. These chimeric mice propagate human prions more “effectively”, and therefore, new chimeric transgenic lines are generated to further reduce the incubation times. This is achieved by altering the sequence similarity of chimeric PrP, by reducing human, and by increasing murine sequences. The shorter incubation time of MHu2M models renders them particularly suitable for bioassays of human prion infectivity and strain properties, but the main limitation is that they poorly recapitulate other aspects of human prion disease.

### Conditional knock-out/knock-in models: the Cre-loxP system

The use of the *Cre/loxP* recombination system enables “conditional” induction or inactivation of the gene of interest at a defined developmental time point, in a cell or tissue-specific manner, or both. The *Cre*-*loxP*-mediated knock-in gene replacement technique was first used in 1996 to generate humanised PrP mice [82] (Fig. 2; Supplementary Table 1) and has since been widely utilised. In addition to the generation of conditional knock-out [154] or knock-in mouse models [18], the *Cre*-*lox* system has been used in other modifications, for example, to generate mice with conditionally expressed soluble dimeric PrP to study the PrP^C^–PrP^Sc^ conversion process [121] [Fig. 4(11)]. A particularly successful model is the transgenic mouse expressing multiple copies of *floxed* (“**fl**anked by ***Lox*** *P* sites”) *Prnp* (Tg37 and Tg46) [107, 108], where upon Cre-mediated recombination, the transgenically expressed PrP is excised at the age of 8–12 weeks. This is achieved by intercrossing Tg37 or Tg46 lines with mice expressing Cre recombinase under the control of the neurofilament heavy chain promoter (NFH–Cre/Tg37 and NFH–Cre/Tg46). The fairly precise onset of Cre expression made this a highly successful model for studying the role of PrP^C^ to understand the role of PrP^Sc^ toxicity [107, 195] and to evaluate potential therapeutic compounds [125, 126, 195].

### Modelling the physiological and pathophysiological functions of PrP: expression of full length, partially deleted or tissue-specific PrP

Following the generation of the *Prnp* ^*0/0*^ mouse [28], a logical and scientifically important experiment was the reintroduction of PrP expression by transgenesis [53] (Fig. 2). The reintroduction of full-length PrP into *Prnp* ^*0/0*^ mice proved the restitution of the propagation of prion disease. Mice expressing full-length PrP were designated Tga20 (the letters “a” to “d” in the original publication indicate increasingly shortened *Prnp* open reading frames). Inoculation of Tga20 mice with RML prions resulted in very short (approximately 60 days) incubation times and a very short disease duration (of only one or two days as compared to a more protracted course of the disease in wild-type mice expressing two copies of *Prnp*). In contrast, heterozygous mice expressing only one allele of *Prnp* (*Prnp* ^+*/0*^) [53] had a significantly prolonged incubation times and a comparatively mild clinical course. Tga20 mice proved extremely popular for transmission experiments due to the very short incubation time for studies on neurotoxicity and prion spread within the brain or neural pathways into the brain [22, 23] and for studies of the kinetics of infectivity and prion protein accumulation [157, 158]. Other models overexpressing PrP instead showed neurodegeneration and neuromuscular impairment [193].

### Mutants of PrP: deletions and point mutations

In addition to the re-expression of full-length PrP, Fischer et al. [53] generated multiple amino-terminal deletion mutants of the *Prnp* open reading frame to understand the function of PrP and to determine the minimum requirement for prion replication. These in-frame deletions were of progressive length (Δ32–93, Δ32–106, Δ32–121, and Δ32–134). This led to rather unexpected results, with a remarkable phenotype of progressive cerebellar degeneration in the Δ32–121 and Δ32–134 lines [165] [Fig. 4(9, 11)]. It was speculated that this phenotype could have been caused by the formation of hetero-dimers with full-length PrP [15] and of the Doppel protein interacting with the truncated PrP. However, no human disease corresponding to this intriguing phenotype has been identified yet.

The aim of introducing point mutations into the *Prnp* open reading frame is to generate models of human IPD. An in-frame 144 basepair insertion into the open reading frame of the *PRNP* gene (6-octapeptide repeat insert, OPRI) in familial CJD [132] and the linkage of PrP missense variant, P102L, to the GSS syndrome were reported as early as 1989 [67]. A single amino-acid substitution in the mouse *Prnp* gene (101L, corresponding to the human 102L mutation) was the first model of an IPD (Fig. 2). These mice showed a spontaneous neurological disease, and neuropathological findings were spongiform degeneration and gliosis similar to human disease [70], but critically, no amyloid plaques were formed, in stark contrast to the characteristic neuropathological phenotype in humans. Since then, technological advances have made it possible to create increasingly sophisticated model systems, as for example, the introduction of double replacement gene targeting strategy to generate multiple mouse strains with subtle PrP alterations to understand molecular pathomechanisms of inherited prion diseases and the function of anchorless PrP [37].

### Ectopic and tissue-selective expression of PrP [Fig. 4(2, 3, 4)]

Most models expressing PrP ectopically were generated to understand the function of PrP in a cellular or tissue context, i.e., to study in which cellular or organ system PrP can replicate and propagate infectivity, and eventually cause clinical or subclinical disease. Propagation of hamster prions selectively in astrocytes, but not in neurons was sufficient to render mice susceptible to prion disease [152] [Fig. 4(2)]. Transgenic mice expressing PrP on lymphoid cells (in *Prnp* ^*0/0*^ mice) led to the transmission of infectivity from the spleen into the brain that carried “sentinel” grafts derived from PrP-expressing neural stem cells [20]. More complex reconstitution experiments [85] in turn demonstrated that PrP expression on lymphoid cells is dispensable to propagate prions. Ectopic expression of PrP on oligodendrocytes [144] [Fig. 4(3)] did not help propagating infectivity, whilst expression of truncated forms of PrP (or of Doppel protein) selectively in oligodendrocytes showed myelinotoxicity [151], not a surprising finding, given that this protein also causes Purkinje cell death [154]. Selective expression of full-length PrP in the axon was shown to be required for myelin stability in the peripheral nervous system [24] [Fig. 4(4)].

### Transgenic mice expressing PrP sequences of non-human species

A wealth of transgenic mouse lines has been created expressing PrP sequences of species other than human. The first transgenic mouse containing Syrian golden hamster PrP was reported in 1989 ([163]. Since then, mouse models expressing bovine, ovine, porcine, guinea pig, elk, and bank vole PrP sequences have been made—available for research of transmissible spongiform encephalopathies of both humans and animals. Likewise, increasing numbers of intercrossed lines have been generated to investigate the role of immune system and microglia [Fig. 4(1)] in prion disease pathogenesis [167, 196]. Transgenic mouse lines of other model systems, particularly those related to immune system [24] and cellular processes [115], have shed light on important aspects of prion biology, but a comprehensive review of these models would be beyond the scope of this article.

### Nematodes and flies

Unlike in research into other neurodegenerative diseases, invertebrate models, such as the nematode *C. elegans* and the fly *Drosophila melanogaster*, have so far been utilised very little in prion research. However, recent studies suggest that these models hold a potential for studying, among other aspects, prion neurotoxicity [178], infectivity [177], and testing anti-prion compounds of sporadic and inherited prion disease [56].

## Alternatives to animal models

### Ex vivo models: organotypic slice cultures

Ex vivo experiments are performed on tissue taken out from live organisms, and transferred into an artificial environment. Organotypic slice culture is a popular ex vivo model and the first such assay for studying prion amplification and titration was published in 2008 [51, 52]. Since then, this model system, in particular murine cerebellar organotypic cultured slices, has been used to study the physiological processes involved in prion formation, PrP^C^–PrP^Sc^ interactions, prion-induced neurotoxicity, and to test efficiency of novel therapeutics. Organotypic slice cultures hold potential to bridge the gap between often highly complex in vivo and simpler in vitro models. However, the advantage over in vivo mouse models which provide a much more complete picture on neuropathology, biochemistry, and behaviour has yet to be demonstrated. Even though initiatives to promote “3R” (refinement, reduction, and replacement of animal research) are supportive of such studies, the complexity of culture conditions to maintain brain tissue slices, lack of standardisation, and a potentially difficult readout may have prevented a wider adoption in neurodegeneration research.

### In vitro assays: a versatile, scalable, and reproducible alternative to animal models

Cell-based in vitro models are instead widely used in the research on the cellular biology of prion propagation, alone or in combination with in vivo experiments. The greatest benefits of cell culture models, in comparison with animal studies and organotypic slices, are low costs and experimental simplicity, i.e., reproducibility, standardisation of culture conditions, and experimental readout. The successful infection of the mouse neuroblastoma cell line N2a with RML/Chandler inoculum was reported in 1987 [150], and since then, a wide range of sub-clones have been developed and characterised, of which the PK1 and R33 lines are arguably most commonly used. At least 15 different other animal-derived neuronal and non-neuronal cell lines, including sub-clones, have been developed over years, and each of which shows a distinct susceptibility to prion strains, which on the other hand prevents direct comparison between lines (for review, see [105, 180]). As always, the simplicity and versatility of these assays have a downside: whilst cell culture studies have contributed enormously to the understanding of the mechanisms governing prion biology, many of these findings are not directly relevant to human disease. It is indeed a disappointment that many of the anti-prion compounds, which have been effective in vitro, have failed in subsequent in vivo studies.

### Cell-free assays, purified recombinant prion proteins, and synthetic PrP fragments: from the principle of protein folding to the development of assays

Since the first report of cell-free formation of protease-resistant prion protein in 1994 [94], multiple tools and protocols have been developed to synthetise and purify recombinant mammalian PrP [102]. The most commonly used system for the synthesis of prion protein is *Escherichia coli*. Recombinant PrP of various species (human, mouse, bank vole, and Syrian hamster) has been generated, and many of these assays include deletions and truncations of PrP. Human recombinant PrP has been generated at full length [residues 23–231 (unglycolysated and lacking the GPI anchor)], and with various modifications, such as the expression of the globular domain only (residues 121–231 or 90–231), amino-terminal flexible tail residues (23–120) [98], and Y145Stop (23–144) [99]. The objective of generating synthetic PrP is to study the physiological function of PrP, its role in disease pathogenesis, and conformational properties. Whilst the formation of prions from recombinant PrP in a cell-free system has been demonstrated by some groups [102], a robust approach to reproducibly and systematically generate infectious prions from recombinant PrP in vitro has yet to be developed [161]. The systematic studies of conditions that lead to in vitro aggregation have also aided the development of cell-free assays for the detection of prions. It is beyond the scope of this review to discuss this in detail. Three major technologies, reviewed elsewhere in great detail [10, 162, 192], are further discussed in the following.

### Computer modelling and bioinformatics approaches

Advances in structural studies on molecules and computational modelling are the basis for the development of in silico molecular docking simulation methods, which allow analysing the interaction—binding modes and binding affinity—between various molecules. Thus, in silico computer simulation technique is emerging as a powerful tool for screening novel therapeutics, including anti-prion compounds [63, 133]. It is predicted that the use of in silico virtual screening platforms for the identification of anti-prion compounds will continuously increase.

## Model systems in the context of specific biological questions

In this section of the review article, the focus will be on models that could not be adequately discussed above. This is particularly relevant for crosscutting themes, and includes models which address specific biological questions or mechanisms.

### Identification of the transmissible nature of the disease-causing agent of scrapie and human prion diseases of different aetiologies (Fig. 1)

The transmissible nature of human prion diseases was first demonstrated in non-human primates with the transmission of kuru [54] and sCJD in 1968 [57]. These transmission studies were encouraged by earlier studies of sheep scrapie. Whilst the transmissible and infectious nature of scrapie was long suspected, experimental studies were successful only in 1936 when brain and spinal cord extracts from diseased sheep were transmitted into sheep or goat and the recipients developed scrapie after a 2 year incubation period, confirming its transmissible nature [48, 49]. These experiments were successfully reproduced a few decades later by Pattison [138]. Following the characterisation of the transmission of kuru, it became increasingly apparent that the agent, previously thought to be a slow virus, harboured rather unusual biological properties. These studies were carried out in primates [13, 54, 57] and led to the proposition of the protein-only hypothesis [61] with its subsequent experimental verification in hamsters and mice [21, 146, 147]. In comparison, the proof that vCJD is caused by BSE was established significantly faster, due to the availability of humanised transgenic mice [26, 64] also providing a significant additional insight into the pathogenesis of vCJD [45]. The transmission properties of prions derived from various inherited prion diseases have been extensively studied in animal models, such as primates, inbred wild-type mouse strains, transgenic mice, and bank voles (Fig. 1) [4, 25, 44, 110, 118, 128, 140, 141, 172, 173, 175]. Prion transmission into knock-in humanised mice expressing PrP at various levels and carrying M129 or V129 alleles has been important in deciphering the basis for two distinct neuropathological and biochemical patterns of dural graft-related iCJD. These studies demonstrate that the observed neuropathological differences in these patients are best explained by their infection with distinct sCJD prion strains, and suggest that the human cadaveric dural graft pools may have been contaminated with prions from more than one donor [89]. More recently, the transmission of a newly identified form, variably protease-sensitive prionopathy (VPSPr), has also been confirmed in humanised mouse models [50, 129]. The observed extremely low transmission levels in one of the studies suggest that VPSPr has transmission properties distinct from other prion diseases, such as sCJD and vCJD [50].

### Models to understand species barriers

First evidence of an interspecies transmission barrier of prions and an experimental approach how to overcome it was demonstrated in 1961 when mice developed prion disease following inoculation with brain extracts from goats that had been inoculated with isolates from scrapie sick Suffolk sheep [32]. Following the primary transmission, which showed a species barrier, subsequent passages were 100% effective, with mouse strain specific incubation times [31]. A transmission barrier is defined as an incomplete penetrance of the disease with prolonged incubation periods compared to the host [137]. The species (or transmission) barrier has been extensively modelled in transgenic humanised mice expressing either full-length human or chimeric human and mouse PrP genes (Figs. 1, 2). In vivo and in vitro studies have revealed that both the species-specific primary sequence homology of PrP (that is, differences in amino-acid sequence between host PrP^C^ and donor PrP^Sc^) and prion strain conformation are the major factors determining the species barrier [41]. To circumvent the species barrier and thus facilitate transmission of CJD, a number of different lines of transgenic mice have been produced. Mouse models expressing chimeric mouse–human prion protein transgenes have been particularly important in demonstrating the significance of the primary sequence of PrP to abnormal prion protein cross-seeding efficacy from different species. The relevance of sequence homology has subsequently also been shown in in vitro conversion assays [17, 95] and in experiments with scrapie-infected neuroblastoma cell lines [145]. The significance of prion strain conformation for interspecies transmission barrier as opposed to merely PrP sequence homology is highlighted in studies on yeast prions [171, 176], in study with recombinant PrP from various species [76] and more recently in studies with bank voles [128]. Substitution of a single amino acid in a critical region can be sufficient to change the species specificity and thus remove a species barrier as shown in studies with purified recombinant PrP23–144 of human, mouse, and Syrian hamster, where assets at position 138 and 139 are determinants of species-dependent seeding specificity [181].

Transgenic mice overexpressing human PrP on the background of *Prnp* ^*0/0*^ mice have been widely used to modulate and, when necessary, eliminate the species barrier when transmitting common animal prion diseases, such as BSE, chronic wasting disease, (CWD) and scrapie. Whilst the zoonotic potential of BSE has been unequivocally confirmed through transmission into transgenic mice expressing bovine, chimeric or human sequences [6, 64, 164], a number of studies using various different mouse models overexpressing human PrP on the background of *Prnp* ^*0/0*^ mice have demonstrated lack of such a potential for scrapie and CWD [96, 149, 159, 170]. More recent studies, however, utilizing PMCA in the same model (Tg440, overexpressing human PrP 129M) show that cervid PrP^Sc^ can, in fact, induce the conversion of human PrP^C^ after the cervid PrP^Sc^ strain has been adapted by successive passages in vitro or in vivo [11]. In addition, the zoonotic potential of scrapie has recently been demonstrated in cynomolgus macaques following a 10-year incubation period [46] and in mice overexpressing human PrP [Tg340 (M129) and Tg361 (V129)] [30]. Whilst in mice, the efficiency of transmission of the primary passage was low, subsequent serial passages of different scrapie isolates resulted in higher attack rates and the propagation of prions which are phenotypically indistinguishable to sCJD strains (MM1 and VV2, respectively; Parchi classification, see also Fig. 3 for homologisation with the London classification) [30]. This strain shift of the BSE/vCJD strain towards a human phenotype (i.e., sCJD) inevitably has potential public health implications [7], in that exogenous (i.e., potentially zoonotic) prions adapt towards, and may become indistinguishable from human strains, as shown also for CWD [11] or scrapie [30].

### Prion strains

An enormous advantage of modelling prion diseases over any other neurodegenerative disorder is the ease of inducing it through infection with prions, most commonly by intracerebral inoculation. Prions differ in their conformational states, which forms the basis of the general model of distinct prion strains [41]. In addition to well-characterised strains of sporadic, acquired and inherited human prion diseases, those derived from hamster prions (Sc237 and 263K), mouse-adapted BSE prions (301C and 301V), mouse-adapted scrapie prions, (RML, ME7, 139A, and 79A), and anchorless prions derived from Tg44 mice (22L) have been extensively used to study human prion disease. A widely used animal strain into the research of both human and animal prion diseases is a mouse-adapted sheep scrapie isolate. It is derived from the prion strain RML, which was first described in 1961 by Chandler, and therefore, sometimes is also referred to as Chandler strain [32].

Distinct human prion strains, such as sCJD, vCJD, kuru, iCJD, VPSPr and sporadic Fatal Insomnia, not only differ in their biochemical and neuropathological patterns, but also have characteristic transmission and propagation properties which have been extensively studied in transgenic humanised mouse models [3, 18, 19, 90, 111, 122, 129, 183, 186]. The knowledge gained through these studies is important particularly from a public health point of view, as this can help identifying and characterising the origins of any new atypical strains. For example, transgenic mouse studies have shown that V2 PrP^Sc^ infection of a mouse with 129MM genotype (strain classification according to the Parchi classification [136], see also Fig. 3 for homologisation with the London classification) generates an unusual PrP^Sc^ with altered conformational properties, which retains the “memory” of V2 PrP^Sc^ and re-emerges and replicates rapidly when transmitted to a mouse with a 129VV genotype. Such so-called trace-back studies are thought to be a reliable means for identifying the origin of prion strains [93]. Furthermore, based on the similarities between neuropathological and biochemical features of 129MM mice infected with the V2 PrP^Sc^ strain and those seen in one type of dural graft iCJD, it has been suggested that the combination of *PRNP* 129MM genotype, kuru plaques, and a specific PrP^Sc^ biochemical pattern may indicate an acquired aetiology of CJD [91–93].

### Genetic susceptibility: models of* PRNP* gene polymorphisms and mutations

The human *PRNP* gene encodes methionine (M) or valine (V) at residue 129, which has a strong effect on the susceptibility to human prion diseases [119, 123]. In northern Europe 38% of the population are MM, 51% MV and 11% are VV. Sporadic CJD occurs most frequently in MM homozygous individuals and it is also relevant in the acquired forms of CJD, most strikingly in vCJD, where most clinical cases studied so far have been homozygous for methionine at codon 129 of *PRNP*. A polymorphism at codon 127 (G127V) of *PRNP* has recently been determined as a resistance factor against the kuru epidemic and this observation also dismissed speculations of possible pathogenic mutation that could have triggered the kuru outbreak [120]. In fact, it demonstrates that the polymorphisms at codon 127 and 129 of *PRNP* are a population genetic response to an epidemic of prion disease and can be regarded as a powerful episode of recent selection in humans [120]. Another polymorphism, occurring in Asian populations, with an allele frequency of 6%, is E219K, which has been reported to have a protective effect for sCJD and it influences the clinical–pathological features of GSS with P102L mutation [66].

The importance of the polymorphisms as a disease modifier is also highlighted in the context of pathogenic mutations of the *PRNP* gene, associated with IPD. More than 40 different mutations have been described and comprise changes of the number of octapeptide repeats in the N-terminal domain, PrP missense mutations causing an amino-acid variant in most cases or rarely a premature stop codon [14]. The most common mutations are octapeptide repeat insertions and the point mutations P102L, D178N, E200K, and V210I [14], respectively. IPD comprises a range of heterogenous clinical presentations and neuropathological patterns and represent approximately 15% of all prion diseases (for a summary of genotype–phenotype correlations, see [116]).

To understand the profound influence of common polymorphisms at residue 129 [6, 7, 183], the rare polymorphism at residue 127 [9] and residue 219 [66] of the *PRNP* gene in susceptibility to acquired, sporadic and some inherited prion diseases and association of different polymorphisms with certain prion strains, transgenic, and knock-in humanised mouse models have been of particular importance. A comprehensive list of mouse models of human polymorphisms is given in Supplementary Tables 1 and 2. Studies in mice expressing different combinations of 129M and 129V have been fundamental to understand the contribution of this polymorphism to susceptibility of different human prion strains. These models suggest that individuals with *PRNP* 129MV may be more susceptible to infection with vCJD prions than to cattle BSE prions and may present with a neuropathological phenotype distinct from vCJD [7]. BSE transmission into PrP 129VV mice caused clinical disease with no detectable PrP^Sc^, a phenotype that is also seen in clinically affected BSE-challenged wild-type mice. Thus, BSE and vCJD prion infection in transgenic mice can result in the propagation of distinct molecular and neuropathological phenotypes dependent on host PrP residue 129 [7]. These data predict a critical role for *PRNP* codon 129 in governing the thermodynamic permissibility of human PrP^Sc^ conformation that can be interpreted within a conformational selection model of prion transmission barriers [183].

The recent mouse model for the protective effects of the V127 allele (which is seen only in combination with the M129 *PRNP* allele) [9] elegantly confirms that mice expressing the PrP V127 variant (on one allele, e.g., V127M129; G127M129) are unable to propagate kuru and sCJD prions, but they do propagate vCJD prions. Instead, mice homozygous for human PrP V127 (i.e., V127M129; V127M129) amazingly show a complete resistance to kuru, vCJD, sCJD, GH-iCJD, and DG-iCJD strains. It is obvious that there is a significant potential for further structural studies and eventually to develop effective drug interfering with prion replication.

The E219K polymorphism, prevalent in Asian populations and associated with a partial resistance to sCJD, has been replicated in a knock-in mouse model, which demonstrated that the PrP 219K molecule is readily converted to PrP^Sc^ after challenge with vCJD and that the conversion occurs even more effectively than in the 219E molecule. However, heterozygous 219E/K mice (“mismatch”) showed the least effective conversion, indicating so-called “heterozygous inhibition” [66]. With mismatching alleles, attack rates were much lower and incubation periods longer. Based on these findings, the “conformational selection” hypothesis has been proposed, where the transmission of infectious prions with shorter incubations periods is facilitated if the host prion protein can readily adopt the preferred conformation associated with the strain of the infected prion [40, 41, 182].

### Models of* PRNP* gene mutations (Fig. 5): more promises than they can deliver?

**Fig. 5:**
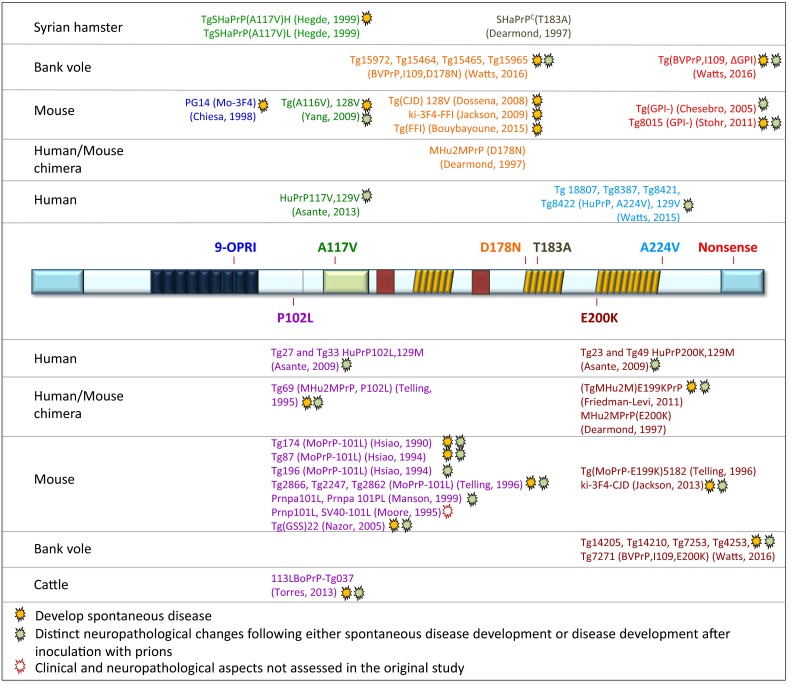
Transgenic mouse models of inherited prion disease. The open reading frame of the *PRNP* gene is represented in the centre, and the mutations that have been modelled in transgenic animals are shown *above* and *below*. On the *left,* the species PrP sequence of the transgene is shown, i.e., hamster, bank vole, mouse, and bovine sequences as well as chimeric constructs, such as human/mouse. The *amber symbols* next to the mutant indicate that the model developed a spontaneous disease and the *green symbol* indicates that there was a distinct neuropathological phenotype either spontaneously or following inoculation with prions. In one study, the clinical and neuropathological aspects were not assessed (*open symbol*). The mouse lines with no symbol did not develop spontaneous disease and did not show distinct neuropathology *Blue* or *red letters* are simply for orientation, to align with the point mutation shown in the *centre*

The first genetic confirmation of the *PRNP* P102L mutation causing GSS in 1989 in two pedigrees [67] prompted the generation of mouse models for further study of the disease mechanism, understanding of phenotypic variability, and transmission properties [68, 70]. Since then, P102L models have been refined [4, 185] although have not always held the promise of full understanding of all aspects of the disease [5] with some results contradicting earlier studies [4]. A number of common point mutations (P102L [4], A117V [8], D178N, E200K [4, 73], as well as octapeptide insertion mutations [74], and mutations associated with formation of anchorless PrP [37]) have been modelled in mice. Whilst the most common approach has been the expression of a transgene on a *Prnp* ^*0/0*^ background, also mice expressing murine, chimeric human/mouse, human, Syrian hamster, bovine, and more recently bank vole PrP sequences have been used. A comprehensive overview of available models expressing pathogenic PrP mutations is given in Fig. 5. With the development of different models, it has emerged that reproducing aspects of human IPD is far more challenging and unsatisfactory than modelling sporadic and acquired human prion diseases. The IPD models have one or more shortcomings: some either do not develop spontaneous disease at all, the disease is not fatal, the attack rates and transmission properties on further passages are variable, or the neuropathology and strain properties do not correspond to human equivalent. For example, the most recently published transgenic mice, generated with an attempt to reproduce human genetic prion disease express bank vole PrP containing corresponding D178N, E200K, and anchorless PrP mutations [187]. Although these mice develop transmissible, highly penetrant spontaneous disease with a neuropathological phenotype (vacuolar degeneration and abnormal PrP immunohistochemically detected deposits) distinct for each mutation, neither anatomical distribution nor neuropathological and biochemical findings correlate well with the human phenotype.

A range of mutant human PrP species (E220K, P102L, A117V, OPRI, D178N 129M, D178N 129V, F198S, and Y163X) has been transmitted into primates, bank voles, and transgenic mice expressing murine, human, or bank vole PrP. Except the truncating Y163X mutation, all other mutant prions are transmissible, but the resulting pathological phenotype does not correspond well to human disease. This discrepancy has recently been reported and discussed in a P102L transgenic mouse model. Contrary to conclusions from the previous studies, 102L mutant prions are transmissible to transgenic mice expressing human PrP carrying the same mutation, but do not infect wild-type mice or mice expressing wild-type human PrP on background of *Prnp* ^*0/0*^ [5].

PrP point mutations directly influence PrP^Sc^ assembly and PrP primary structure significantly influences the phenotype of inherited prion disease. For example, a kindred with substitution at residue 211 (E211D) and individuals with an E211Q mutation, respectively, are reported to have remarkably distinct clinical phenotypes, neuropathology, and biochemical alterations [139]. The E211D mutation was associated with a GSS phenotype clinically and pathologically, whilst the E211Q mutation had a short disease duration and no amyloid plaques. Such strong influence of a single amino-acid substitution on the phenotype has also been confirmed in vitro and in vivo [139] and in vivo [73]. In keeping, phenotypical variations have long been known in families with pathogenic mutations. One explanation is the spectrum of involvement of protease-resistant wild-type PrP, as demonstrated in P102L IPD [185]. This study showed that in patients with P102L mutations, a substantial proportion of abnormal PrP is derived from the wild-type allele.

## Models of the formation, spread, toxicity, different strains of the abnormal prion protein, and cellular pathways

### Bioassays: detection and measurement of infectivity titres

A robust, sensitive, and specific bioassay to detect prion infectivity is undoubtedly highly desirable for clinical diagnostics, epidemiological studies, and laboratory experimental assays. For clinical diagnostic purposes, a bioassay would have to be sensitive, specific, and should be achievable in a comparatively short turnaround time. Therefore, the transmission into animals sensitive for human prion disease is not an option due to the long incubation times, comparatively high costs and ethical reasons. In addition, clinical studies would require validation and suitability of the assay for an accredited environment. Therefore, cell-free assays have the greatest potential for clinical purposes. The two most widely used cell-free assays are Protein Misfolding Cyclic Amplification (PMCA) [34] and Real-Time Quaking-Induced Conversion (RT-QuIC) [113]. PMCA uses PrP^C^ for the amplification of PrP^Sc^ through repeated series of incubation and sonification. RT-QuIC uses automated quaking or shaking for the amplification of PrP^Sc^ from recombinant PrP^C^. Both cell-free-based assays detect PrP^Sc^ with remarkably high sensitivity. The limitations of PMCA are the risk of cross contamination and the generation of de novo PrP^Sc^ by off-target amplification. RT-QuIC is limited by the readout and a non-infective end product , which makes it unsuitable, for example, to study effectiveness of decontamination of anti-prion compounds. A blood based assay, developed for the detection of vCJD in blood, and also of early stages of preclinical disease (Direct Detection Assay, DDA) [160] utilises a solid-state binding matrix to capture and concentrate PrP^Sc^, which is then detected immunologically. It is suitable for high-throughput screening in the surveillance of an at-risk population [72] and for the detection of abnormal PrP conformers in animal models (CD1 mice, PrP^C^ overexpressing mice (Tga20), and Syrian hamsters) [160]. Scrapie cell-based assays (SSCA), based on a cell line derived from N2a cells that are highly susceptible to RML prions, have been in development over several decades [88], and with standardised methodology, they are suitable for automation and high throughput. Their refinement makes it possible to achieve high-throughput isolation of prions even to exceptionally high levels of purity with retained biological and biochemical prion strain properties [192].

Cell-based assays, in combination with animal studies, have been instrumental in understanding the dynamics of prion titres and the accommodation of toxic species in the progression of prion disease and for detailed correlation between neuropathological changes and prion titre [157, 158]. Instead, classical transmission studies by serial dilution of an inoculum and subsequent transmission into large cohorts of animals, popular in the early times of prion research, and gold standard to determine titres in brains or brain regions [22] are becoming increasingly obsolete.

### Formation of PrP^Sc^ and toxic oligomers in vivo

Conversion of PrP^C^ into PrP^Sc^ is a post-translational process, which occurs when PrP^C^ reaches the extracellular domain. PrP^C^ and PrP^Sc^ have identical amino-acid sequences but differ in their secondary and tertiary structures. PrP^Sc^ forms β-sheet-rich aggregates, which range from small, soluble oligomers to large insoluble fibrillary structures. The formation of PrP^Sc^ and the development of neurodegenerative changes require PrP^C^ as a substrate [22]. Prion propagation in the brain involves two phases [157]. In the first phase, prions propagate exponentially andare not rate limited by the concentration of PrP^C^. They rapidly rise and reach a maximal titre. The titre is independent of PrP^C^ concentration, i.e., there is no difference between Tga20, *Prnp* ^+*/*+^ and *Prnp* ^+*/0*^ mice. The flowing plateau phase determines the latency to clinical disease onset. The plateau phase is inversely proportional to the PrP^C^ concentration. These studies [157, 158] demonstrate that propagation and neurotoxicity can be uncoupled, arguing that prions themselves are not directly neurotoxic, supporting claims of previous studies where prions generated by an infected neural graft did not cause degeneration of adjacent *Prnp* ^*0/0*^ host tissue [22]. Uncoupling of accumulated PrP^Sc^ and neurotoxicity was also shown in the model of conditional inactivation of PrP expression in neurons, reversing clinical disease and halting spongiform changes and neuronal loss [107].

### Cellular pathways as a response to prion toxicity

Cellular pathways implicated in misfolded protein response in prion disease can be located on the cellular surface or can be intracellular. PrP^C^ molecules with internal deletions (for example, Δ94–134 and Δ105–125) cause neurodegeneration [165]. The toxicity is more likely caused by abnormal activity on the neuronal surface, for example, interaction with NMDA receptors [77] rather than abnormal folding (discussed in detail in [38]). More relevant to the toxicity of misfolded proteins are intracellular pathways, the ubiquitin–proteasome system, and endoplasmic reticulum (ER). Early functional impairment of the ubiquitin–proteasome system to degrade infectious prions has been shown in cell-based in vitro studies [103], in vivo in flies [127], and in transgenic mouse models of the ubiquitin–proteasome system [Fig. 4(6)] [115]. In the conditional knock-out Tg37 mouse line and in wild-type mice, accumulation of PrP^Sc^ causes sustained over-activation of the PERK/eIF2alpha branch of the cellular defence pathway—unfolded protein response (UPR) with phosphorylation of eIF2alpha [Fig. 4(10)]. This leads to sustained repression of global protein synthesis (translational repression), with subsequent synaptic failure and neuronal loss [126]. The discovery of the involvement of these pathways in prion disease provides a scientific rationale for the pharmacological enhancement of the ubiquitin–proteasome system and to target the ER unfolded protein response pathway in prion disease.

### Models to understand the role of the immune system in the spread of prions

Whilst sporadic forms of prion disease have their origin within the CNS, acquired forms can involve the lymphoreticular system. The transmission of BSE prions through the food chain caused vCJD in humans. A highly distinctive and diagnostic feature of this prion strain was the colonisation of the lymphoreticular system [65], and this has generated widespread interest into the role of the immune system in prion diseases. Prior to the outbreak of vCJD, however, the role of the spleen in modulating neuro-invasion had been studied in scrapie-infected mice [81], where spleen removal reduced efficiency of peripheral prion infection, but had no effect once the infection had reached the CNS. The extensive use of transgenic mice, ectopically expressing prion protein on the lymphoid system or genetically modified immunosuppressed mice, has further pinpointed the role of individual components of the lymphoreticular system in prion spread [84, 85]. It is thought that prions invade the central nervous system from lymphoid organs by ascending spread via sympathetic and parasympathetic nerves [60, 112] and that anchored PrP is an essential component for this pathway [86]. For more detailed reviews on the lymphoid system in prion spread, see [43] and [1]. The importance of the immune system has been successfully shown in preclinical studies with anti-PrP antibodies [194].

### Models to investigate potential therapeutic approaches

The goal of biomedical research is to understand, prevent, and eventually treat human disease. This is no different for prion diseases, and developing effective treatments of prion diseases is a priority for many research teams. Clinical trials have to be evidence-based, and in vitro and in vivo models are essential to identify and characterise pathways that can be therapeutically targeted or can be used to validate “anti-prion compounds” in preclinical studies. However, the beneficial effects of compounds or reagents have not yet translated into effective clinical trials in patients [42, 62], but at least have led to important quantitative clinical data.

*In silico* computational modelling in combination with cell-based screening systems [71] can be the first-line experimental approach to identify potential anti-prion therapeutics. This is usually further validated in animal models, where prion infected mice are challenged with promising candidate drugs [63, 125].

The use of monoclonal antibodies has been promising in preventing prion spread and in attenuating and delaying clinical prion disease [194]. These studies were carried out in wild-type mice of a defined genetic background. The safety of the use of monoclonal antibodies, however, has been a matter of considerable debate. Whilst toxicity of concentrated monoclonal antibodies was reported by some researchers [153, 166], others could not reproduce these findings [87], in particular when using physiological concentrations. A widely used transgenic model to assess therapeutic efficacy is the Tga20 mouse line that overexpresses wild-type PrP. For example, in a recent study [63], Tga20 mouse line was used to identify, through iterative cycles of chemical design and synthesis, luminescent conjugated polythiophenes (molecules with high affinity for ordered protein aggregates) as potentially effective anti-prion compounds.

The conditional knock-out mouse line Tg37 [107] was used in a study into the effect of a reduction of PrP^C^ expression through therapeutic knockdown using RNAi in mice with established prion disease [195]). This model was also used to show positive effects of UPR inhibition by localized hippocampal PrP knockdown and through pharmacological inhibition with the compound GSK2606414 of PERK kinase, a key mediator of the UPR pathway in the endoplasmic reticulum [125, 126]. The compounds targeting the cellular downstream pathways—for example, PERK kinase inhibitor GSK2606414 causes severe toxicity in mice, making it unlikely that it will make it to a clinical trial in humans.

Given that innovative genetic strategies to halt or reverse progression of neurodegenerative diseases are emerging, it is possible that genetic manipulation with the end-result mimicking naturally occurring protective/prion-resistant single amino-acid substitutions may be a treatment of choice in prion diseases, particularly those of inherited aetiology. Validation and approval of such approaches undoubtedly will require extensive detailed in vitro cell-based and in vivo animal studies in existing and newly generated model systems of prion infection.

## Summary

The development and characterisation of models of prion disease have served a wide range of purposes, which have changed over time. The first models were used to understand the transmissible nature of prions, well before even the concept of transmissible proteopathies was born. These models date back to the 1930s [47, 48] and culminated in the demonstration that kuru and CJD are transmissible neurodegenerative diseases in the 1960s [13, 54, 55], leading to the hypothesis that scrapie is caused by an unusual self-replicating agent [61]. Nearly two decades later, using a combination of transmission studies and biochemical evidence, Prusiner formulated the protein-only hypothesis [21, 114, 146]. The availability of genetically engineered mice in the 1980s then opened a wider area of research [68, 148] in that functional aspects of PrP and human mutations were modelled. The use of transgenic mice in prion research took-off after the generation of a mouse model devoid of PrP^C^, generated by Weissmann’s team [22, 27, 28, 155]. This model enabled the generation of mice devoid of endogenous PrP and was the basis for systematic modelling structural and functional aspects of PrP [20, 22, 53, 190, 191]. These mice were far superior to any previous model system to understand prion strains [2, 45], transmission barriers [184], tissue-specific effects of normal and abnormal PrP [59, 143, 144], the role of deletions, point mutations and modifications [165] affecting structure, and glycosylation [37, 86, 142]. In parallel, powerful cell-based and cell-free in vitro systems have been developed [88, 160] and complemented the animal models. As pointed out in the paragraph on therapeutic approaches, the ultimate goal of model systems is to understand, prevent, and treat human prion diseases. Thus, the model systems described in this review are only the beginning of the discovery phase. Whilst these model systems over the decades have become increasingly validated (through the use in several laboratories), they still have to live up to the promises of providing a robust and valid experimental platform that can be used for drug discovery which translates well into a clinical trial.

## Perspective (Fig. 6)

Studies with animal models and cell culture will most likely remain the mainstay in prion disease research for foreseeable future. The use of rat models has been proposed due to advantages related to larger brain size and physiology, including cognitive and behavioural responses somewhat closer to human. However, the greatest potential undoubtedly lies in mouse models. These have proven to be invaluable in prion disease research and it is likely that further models will be able to address the increasingly complex questions of prion biology.

**Fig. 6:**
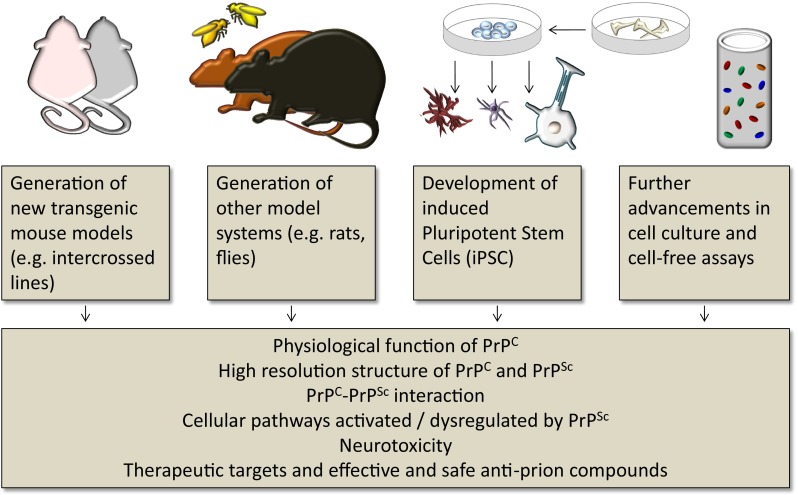
Future perspective in prion disease research: *left* increasing sophistication of transgenic models, and increasing access to gene editing methodologies is likely to have a significant role also in prion research. *Left centre* other species, in particular those which are easy to maintain and to manipulate, such as flies may play a role in models, in particular aiming at functional roles of PrP; increasing use of rat models due to the larger brain size. *Centre-right* a significant potential of induced pluripotent stem cells can be expected, and *right* in vitro cell-based and cell-free assays will play an important role in diagnostics, epidemiology, and safety of blood products and drug screening

The discovery of a comparatively straightforward methodology to induce pluripotent stem cells (iPSC) through reprogramming of fibroblasts (Takahashi and Yamanaka 2006) opened new avenues for the study of cellular pathomechanisms, early disease diagnosis, and screening of therapeutic compounds in probably almost all human diseases. Misfolded protein-associated neurodegenerative disorders, such as Parkinson’s disease and Alzheimer’s disease, are diseases, where iPSC derived from fibroblasts from skin or dura mater have been already actively used in research over the last decade providing novel insights into the molecular biology. Application of the reprogrammed cells in prion research, along with continuous advances in the somatic cell reprogramming techniques, offers a promising tool for deepening the understanding of the molecular biology of prion infection, such as susceptibility, modes and sites (neurones or glia or both) of prion replication, neurotoxicity, effects on cellular function, disease phenotypes and new drug screening, or, indeed, iPSC could serve as therapeutic agent themselves. Finally, significant resources are put into advancing technological and methodological aspects of designing cell-free assays and high-throughput in silico screening methods. The power of bioinformatics and computational chemistry holds promises for the development of high-throughput in silico screening methods, but also are an important development to support the commitment to replacement, reduction, and refinement of animal research.

## Electronic supplementary material

Below is the link to the electronic supplementary material.

Supplementary material 1 (XLSX 22 kb)

Supplementary material 2 (DOCX 89 kb)
