# Supplementary material for: Prion disease: experimental models and reality
Source: Acta Neuropathol. 2017 Jan 13;133(2):197–222. doi: 10.1007/s00401-017-1670-5 (PMC5250673; doi:10.1007/s00401-017-1670-5)
Supplement: Supplementary file 2 — Supplementary material 2 (DOCX 89 kb) [file 401_2017_1670_MOESM2_ESM.docx]

**Table 2:** Overview of common published mouse models of prion disease. The table is grouped by the type of model, with the published designation and the corresponding human polymorphism. The upper part of the table lists mice expressing humanised/human PRP sequences, but excludes models of pathogenic human mutations. The lower part of the table lists mouse models expressing full-length mouse PrP. The right column indicates published studies using these models.

| **Humanised mice on *Prnp*^0/0^ background** | | **First reference** | **Studies carried out on the mouse line** |
| --- | --- | --- | --- |
| Tg110 | Hu(V129) | (Telling et al., 1994) | - Comparison of transmission properties between humanised PrP and chimeric human-mouse PrP (Telling et al., 1994) |
| Tg152 | Hu(V129) | (Telling et al., 1994) | - Comparison of transmission properties between humanised PrP and chimeric human-mouse PrP (Telling et al., 1994) - Susceptibility to BSE in humanised mice (Collinge et al., 1995) - Identification of the same prion strains in vCJD and BSE (Hill et al., 1997) - Comparison of incubation times between humanised PrP and chimeric human-mouse PrP (Korth et al., 2003) - Transmission properties of inherited prion disease due to 6-OPRI mutation (Mead et al., 2006) - Kuru prions have equivalent transmission properties with sCJD prions (Wadsworth et al., 2008) - No transmission of chronic wasting disease (Sandberg et al., 2010) - Prion strain resistance upon repeated exposure to therapeutic compounds (Berry et al., 2013) - Transmission properties of inherited prion disease due to Y163X truncation mutation (Mead et al., 2013) |
| Tg35 | Hu(M129) | (Asante et al., 2002) | - Neuropathological and molecular phenotypes following central infection with BSE and vCJD prion strains in M129 homozygous mice (Asante et al., 2002) - No transmission of chronic wasting disease (Sandberg et al., 2010) - Transmission properties of inherited prion disease due to Y163X truncation mutation (Mead et al., 2013) |
| Tg45 | Hu(M129) | (Asante et al., 2002) | - Neuropathological and molecular phenotypes following central infection with BSE and vCJD prion strains in M129 homozygous mice (Asante et al., 2002) - No transmission of chronic wasting disease (Sandberg et al., 2010) |
| Tg45/152 | Hu(MV129) | (Asante et al., 2006) | - Neuropathological and molecular phenotypes following infection with BSE and vCJD strains in PrP129 heterozygous mice (Asante et al., 2006) |
| Tg440 | Hu(M129) | (Telling et al., 1995) | - Comparison of incubation times between humanised PrP and chimeric human-mouse PrP (Korth et al., 2003; Telling et al., 1995) - No transmission of chronic wasting disease CWD (Tamguney et al., 2006) - Cervid PrPSc can induce conversion of human PrPC after CWD strain has been stabilized by successive passages in vitro or in vivo (Barria et al., 2011) |
| Tg1 | Hu(M129) | (Kong et al., 2005) | - Species barrier for transmission of elk CWD to humans (Kong et al., 2005) |
| Tg40 | Hu(M129) | (Kong et al., 2005) | - Species barrier for transmission of elk CWD to humans (Kong et al., 2005) - The human transmission risk of atypical BSE strains (Kong et al., 2008) - Transmission characteristics of variably protease-sensitive prionopathy (Notari et al., 2014) |
| Tg650 | Hu(M129) | (Beringue et al., 2008) | - Assessment of transmission efficiency and strain phenotype in brain and spleen following central and peripheral injections of vCJD and sCJD inocula (Beringue et al., 2008) - Human susceptibility to small ruminants-passaged BSE prions (Padilla et al., 2011) - Transmission efficiency and strain properties of MM2-sCJD intracerebral inoculates (Chapuis et al., 2016) |
| Tg2669 | Hu(M129) | (Berry et al., 2013) | - Prion strain resistance upon repeated exposure to therapeutic compounds (Berry et al., 2013) |
| Tg183 | Hu(V127M129) | (Asante et al., 2015) | - HuPrP V127 resistance to prion disease development (Asante et al., 2015) |
| Tg190 | Hu(V127M129) | (Asante et al., 2015) | - HuPrP V127 resistance to prion disease development (Asante et al., 2015) |
| Tg35c | Hu(G127M129) | (Asante et al., 2015) | - HuPrP V127 resistance to prion disease development (Asante et al., 2015) |
| Tg152c | Hu(G127V129) | (Asante et al., 2015) | - HuPrP V127 resistance to prion disease development (Asante et al., 2015) |
| Tg340 | Hu(M129) | (Padilla et al., 2011) | - Human susceptibility to small ruminants-passaged BSE prions (Padilla et al., 2011) - Zoonotic potential of scrapie prions (Cassard et al., 2014) |
| Tg361 | Hu(V129) | (Cassard et al., 2014) | - Zoonotic potential of scrapie prions (Cassard et al., 2014) |
| Tg66 | Hu(M129) | (Race et al., 2009) | - Lack of transmission of CWD-infected squirrel monkey brain tissue to humanised mice (Race et al., 2009) - The effect of lack of GPI anchoring on mouse-human PrP species barrier (Race et al., 2015) |
| TgRM | Hu(M129) | (Race et al., 2009) |  |
| Tg21 | Hu(V129) | (Notari et al., 2014) | - Transmission characteristics of variably protease-sensitive prionopathy (Notari et al., 2014) |
| Tg40h | Hu(M129) |  |  |
| Tg362 | Hu(V129) |  |  |
| Knock-in V | Hu(V129) | (Bishop et al., 2006) | - Predicting susceptibility and incubation time of human to human transmission of vCJD (Bishop et al., 2006) - Assessment of transmission efficiency and strain type following secondary transmission of vCJD via blood transfusion (Bishop et al., 2008) - Detailed characterisation of sCJD strains to allow identification of atypical and novel strains of human prion diseases (Bishop et al., 2010) |
| Knock-in M | Hu(M129) |  |  |
| Knock-in MV | Hu(MV129) |  |  |
| Ki-Hu129MM | Hu(M129) | (Asano et al., 2006) | - Relationship between codon129 genotype and susceptibility to BSE and vCJD prions (Asano et al., 2006) - Two distinct subgroups of dural graft CJD caused by infection with different sCJD PrPSc strains (Kobayashi et al., 2007; Kobayashi et al., 2014) - Identification of acquired (iatrogenic) CJD amongst presumed sCJD cases (Kobayashi et al., 2015) |
| Ki-Hu129MV | Hu(MV129) |  |  |
| Ki-Hu129VV | Hu(V129) |  |  |

| **PrP overexpression** |  |  |  |
| --- | --- | --- | --- |
| Tga20 |  |  | - Difference in neurology following high and low-dose inoculum (Thackray et al., 2002) - Difference in neurology between Tga20 and wild-type mice after oral administration of RML prions (Thackray et al., 2003) - Two distinct phases of prion propagation and toxicity (Sandberg et al., 2011) - Globular domain and flexible tail modules of PrPC and flexible-tail mediated toxicity (Sonati et al., 2013) - NOX2 as a major source of ROS in prion pathogenesis (Sorce et al., 2014) - Polythiophenes as antiprion compounds (Herrmann et al., 2015) - Neuroprotective role for microglia in prion diseases (Zhu et al., 2016) |
| Tg19 |  |  |  |
| Tg37 | Hemizygous or homozygous | (Mallucci et al., 2002) | - Effects of post-natal PrP depletion on neuronal survival and function (Mallucci et al., 2002) - Effects of neuronal PrPC depletion in prion-diseased mice (Mallucci et al., 2003) - RNAi therapeutics in prion disease (White et al., 2008) - Comparative transcriptomic analysis of wild-type and transgenic mice brain at zygotic and adult stages (Chadi et al., 2010) - UPR involvement in prion disease (Moreno et al., 2012) - Globular domain and flexible tail modules of PrPC and flexible-tail mediated toxicity (Sonati et al., 2013) - Treatments targeting UPR (Halliday et al., 2015; Moreno et al., 2013) - Brainstem pathology in prion-diseased mice (Mirabile et al., 2015) - Structural plasticity and protective effects of cooling in neurodegeneration (Peretti et al., 2015) |
| Tg46 |  | (Mallucci et al., 2002) | - Effects of post-natal PrP depletion on neuronal survival and function (Mallucci et al., 2002) - Effects of neuronal PrPC depletion in prion-diseased mice (Mallucci et al., 2003) |
|  |  |  |  |
| **Post-natal deletion of PrP** |  |  |  |
| Tg37-Cre 22 |  | (Mallucci et al., 2002) | - Effects of post-natal PrP depletion on neuronal survival and function (Mallucci et al., 2002) - Effects of neuronal PrPC depletion in prion-diseased mice (Mallucci et al., 2003) - Brainstem pathology in prion-diseased mice (Mirabile et al., 2015) |
| Tg46-Cre 22 |  | (Mallucci et al., 2002) | - Effects of post-natal PrP depletion on neuronal survival and function (Mallucci et al., 2002) - Effects of neuronal PrPC depletion in prion-diseased mice (Mallucci et al., 2003) |
|  |  |  |  |
|  |  |  |  |

Asano, M., Mohri, S., Ironside, J. W., Ito, M., Tamaoki, N., and Kitamoto, T. (2006). vCJD prion acquires altered virulence through trans-species infection. Biochemical and biophysical research communications *342*, 293-299.

Asante, E. A., Linehan, J. M., Desbruslais, M., Joiner, S., Gowland, I., Wood, A. L., Welch, J., Hill, A. F., Lloyd, S. E., Wadsworth, J. D., and Collinge, J. (2002). BSE prions propagate as either variant CJD-like or sporadic CJD-like prion strains in transgenic mice expressing human prion protein. The EMBO journal *21*, 6358-6366.

Asante, E. A., Linehan, J. M., Gowland, I., Joiner, S., Fox, K., Cooper, S., Osiguwa, O., Gorry, M., Welch, J., Houghton, R.*, et al.* (2006). Dissociation of pathological and molecular phenotype of variant Creutzfeldt-Jakob disease in transgenic human prion protein 129 heterozygous mice. Proceedings of the National Academy of Sciences of the United States of America *103*, 10759-10764.

Asante, E. A., Smidak, M., Grimshaw, A., Houghton, R., Tomlinson, A., Jeelani, A., Jakubcova, T., Hamdan, S., Richard-Londt, A., Linehan, J. M.*, et al.* (2015). A naturally occurring variant of the human prion protein completely prevents prion disease. Nature *522*, 478-481.

Barria, M. A., Telling, G. C., Gambetti, P., Mastrianni, J. A., and Soto, C. (2011). Generation of a new form of human PrP(Sc) in vitro by interspecies transmission from cervid prions. The Journal of biological chemistry *286*, 7490-7495.

Beringue, V., Le Dur, A., Tixador, P., Reine, F., Lepourry, L., Perret-Liaudet, A., Haik, S., Vilotte, J. L., Fontes, M., and Laude, H. (2008). Prominent and persistent extraneural infection in human PrP transgenic mice infected with variant CJD. PloS one *3*, e1419.

Berry, D. B., Lu, D., Geva, M., Watts, J. C., Bhardwaj, S., Oehler, A., Renslo, A. R., DeArmond, S. J., Prusiner, S. B., and Giles, K. (2013). Drug resistance confounding prion therapeutics. Proceedings of the National Academy of Sciences of the United States of America *110*, E4160-4169.

Bishop, M. T., Hart, P., Aitchison, L., Baybutt, H. N., Plinston, C., Thomson, V., Tuzi, N. L., Head, M. W., Ironside, J. W., Will, R. G., and Manson, J. C. (2006). Predicting susceptibility and incubation time of human-to-human transmission of vCJD. Lancet neurology *5*, 393-398.

Bishop, M. T., Ritchie, D. L., Will, R. G., Ironside, J. W., Head, M. W., Thomson, V., Bruce, M., and Manson, J. C. (2008). No major change in vCJD agent strain after secondary transmission via blood transfusion. PloS one *3*, e2878.

Bishop, M. T., Will, R. G., and Manson, J. C. (2010). Defining sporadic Creutzfeldt-Jakob disease strains and their transmission properties. Proceedings of the National Academy of Sciences of the United States of America *107*, 12005-12010.

Cassard, H., Torres, J. M., Lacroux, C., Douet, J. Y., Benestad, S. L., Lantier, F., Lugan, S., Lantier, I., Costes, P., Aron, N.*, et al.* (2014). Evidence for zoonotic potential of ovine scrapie prions. Nature communications *5*, 5821.

Chadi, S., Young, R., Le Guillou, S., Tilly, G., Bitton, F., Martin-Magniette, M. L., Soubigou-Taconnat, L., Balzergue, S., Vilotte, M., Peyre, C.*, et al.* (2010). Brain transcriptional stability upon prion protein-encoding gene invalidation in zygotic or adult mouse. BMC Genomics *11*, 448.

Chapuis, J., Moudjou, M., Reine, F., Herzog, L., Jaumain, E., Chapuis, C., Quadrio, I., Boulliat, J., Perret-Liaudet, A., Dron, M.*, et al.* (2016). Emergence of two prion subtypes in ovine PrP transgenic mice infected with human MM2-cortical Creutzfeldt-Jakob disease prions. Acta neuropathologica communications *4*, 10.

Collinge, J., Palmer, M. S., Sidle, K. C., Hill, A. F., Gowland, I., Meads, J., Asante, E., Bradley, R., Doey, L. J., and Lantos, P. L. (1995). Unaltered susceptibility to BSE in transgenic mice expressing human prion protein. Nature *378*, 779-783.

Halliday, M., Radford, H., Sekine, Y., Moreno, J., Verity, N., le Quesne, J., Ortori, C. A., Barrett, D. A., Fromont, C., Fischer, P. M.*, et al.* (2015). Partial restoration of protein synthesis rates by the small molecule ISRIB prevents neurodegeneration without pancreatic toxicity. Cell Death Dis *6*, e1672.

Herrmann, U. S., Schutz, A. K., Shirani, H., Huang, D., Saban, D., Nuvolone, M., Li, B., Ballmer, B., Aslund, A. K., Mason, J. J.*, et al.* (2015). Structure-based drug design identifies polythiophenes as antiprion compounds. Science translational medicine *7*, 299ra123.

Hill, A. F., Desbruslais, M., Joiner, S., Sidle, K. C., Gowland, I., Collinge, J., Doey, L. J., and Lantos, P. (1997). The same prion strain causes vCJD and BSE. Nature *389*, 448-450, 526.

Kobayashi, A., Asano, M., Mohri, S., and Kitamoto, T. (2007). Cross-sequence transmission of sporadic Creutzfeldt-Jakob disease creates a new prion strain. The Journal of biological chemistry *282*, 30022-30028.

Kobayashi, A., Matsuura, Y., Mohri, S., and Kitamoto, T. (2014). Distinct origins of dura mater graft-associated Creutzfeldt-Jakob disease: past and future problems. Acta neuropathologica communications *2*, 32.

Kobayashi, A., Parchi, P., Yamada, M., Mohri, S., and Kitamoto, T. (2015). Neuropathological and biochemical criteria to identify acquired Creutzfeldt-Jakob disease among presumed sporadic cases. Neuropathology : official journal of the Japanese Society of Neuropathology.

Kong, Q., Huang, S., Zou, W., Vanegas, D., Wang, M., Wu, D., Yuan, J., Zheng, M., Bai, H., Deng, H.*, et al.* (2005). Chronic wasting disease of elk: transmissibility to humans examined by transgenic mouse models. The Journal of neuroscience : the official journal of the Society for Neuroscience *25*, 7944-7949.

Kong, Q., Zheng, M., Casalone, C., Qing, L., Huang, S., Chakraborty, B., Wang, P., Chen, F., Cali, I., Corona, C.*, et al.* (2008). Evaluation of the human transmission risk of an atypical bovine spongiform encephalopathy prion strain. Journal of virology *82*, 3697-3701.

Korth, C., Kaneko, K., Groth, D., Heye, N., Telling, G., Mastrianni, J., Parchi, P., Gambetti, P., Will, R., Ironside, J.*, et al.* (2003). Abbreviated incubation times for human prions in mice expressing a chimeric mouse-human prion protein transgene. Proceedings of the National Academy of Sciences of the United States of America *100*, 4784-4789.

Mallucci, G., Dickinson, A., Linehan, J., Klohn, P. C., Brandner, S., and Collinge, J. (2003). Depleting neuronal PrP in prion infection prevents disease and reverses spongiosis. Science *302*, 871-874.

Mallucci, G. R., Ratte, S., Asante, E. A., Linehan, J., Gowland, I., Jefferys, J. G., and Collinge, J. (2002). Post-natal knockout of prion protein alters hippocampal CA1 properties, but does not result in neurodegeneration. The EMBO journal *21*, 202-210.

Mead, S., Gandhi, S., Beck, J., Caine, D., Gajulapalli, D., Carswell, C., Hyare, H., Joiner, S., Ayling, H., Lashley, T.*, et al.* (2013). A novel prion disease associated with diarrhea and autonomic neuropathy. The New England journal of medicine *369*, 1904-1914.

Mead, S., Poulter, M., Beck, J., Webb, T. E., Campbell, T. A., Linehan, J. M., Desbruslais, M., Joiner, S., Wadsworth, J. D., King, A.*, et al.* (2006). Inherited prion disease with six octapeptide repeat insertional mutation--molecular analysis of phenotypic heterogeneity. Brain : a journal of neurology *129*, 2297-2317.

Mirabile, I., Jat, P. S., Brandner, S., and Collinge, J. (2015). Identification of clinical target areas in the brainstem of prion-infected mice. Neuropathology and applied neurobiology *41*, 613-630.

Moreno, J. A., Halliday, M., Molloy, C., Radford, H., Verity, N., Axten, J. M., Ortori, C. A., Willis, A. E., Fischer, P. M., Barrett, D. A., and Mallucci, G. R. (2013). Oral treatment targeting the unfolded protein response prevents neurodegeneration and clinical disease in prion-infected mice. Science translational medicine *5*, 206ra138.

Moreno, J. A., Radford, H., Peretti, D., Steinert, J. R., Verity, N., Martin, M. G., Halliday, M., Morgan, J., Dinsdale, D., Ortori, C. A.*, et al.* (2012). Sustained translational repression by eIF2alpha-P mediates prion neurodegeneration. Nature *485*, 507-511.

Notari, S., Xiao, X., Espinosa, J. C., Cohen, Y., Qing, L., Aguilar-Calvo, P., Kofskey, D., Cali, I., Cracco, L., Kong, Q.*, et al.* (2014). Transmission characteristics of variably protease-sensitive prionopathy. Emerging infectious diseases *20*, 2006-2014.

Padilla, D., Beringue, V., Espinosa, J. C., Andreoletti, O., Jaumain, E., Reine, F., Herzog, L., Gutierrez-Adan, A., Pintado, B., Laude, H., and Torres, J. M. (2011). Sheep and goat BSE propagate more efficiently than cattle BSE in human PrP transgenic mice. PLoS pathogens *7*, e1001319.

Peretti, D., Bastide, A., Radford, H., Verity, N., Molloy, C., Martin, M. G., Moreno, J. A., Steinert, J. R., Smith, T., Dinsdale, D.*, et al.* (2015). RBM3 mediates structural plasticity and protective effects of cooling in neurodegeneration. Nature *518*, 236-239.

Race, B., Meade-White, K. D., Miller, M. W., Barbian, K. D., Rubenstein, R., LaFauci, G., Cervenakova, L., Favara, C., Gardner, D., Long, D.*, et al.* (2009). Susceptibilities of nonhuman primates to chronic wasting disease. Emerging infectious diseases *15*, 1366-1376.

Race, B., Phillips, K., Meade-White, K., Striebel, J., and Chesebro, B. (2015). Increased infectivity of anchorless mouse scrapie prions in transgenic mice overexpressing human prion protein. Journal of virology *89*, 6022-6032.

Sandberg, M. K., Al-Doujaily, H., Sharps, B., Clarke, A. R., and Collinge, J. (2011). Prion propagation and toxicity in vivo occur in two distinct mechanistic phases. Nature *470*, 540-542.

Sandberg, M. K., Al-Doujaily, H., Sigurdson, C. J., Glatzel, M., O'Malley, C., Powell, C., Asante, E. A., Linehan, J. M., Brandner, S., Wadsworth, J. D., and Collinge, J. (2010). Chronic wasting disease prions are not transmissible to transgenic mice overexpressing human prion protein. The Journal of general virology *91*, 2651-2657.

Sonati, T., Reimann, R. R., Falsig, J., Baral, P. K., O'Connor, T., Hornemann, S., Yaganoglu, S., Li, B., Herrmann, U. S., Wieland, B.*, et al.* (2013). The toxicity of antiprion antibodies is mediated by the flexible tail of the prion protein. Nature *501*, 102-106.

Sorce, S., Nuvolone, M., Keller, A., Falsig, J., Varol, A., Schwarz, P., Bieri, M., Budka, H., and Aguzzi, A. (2014). The role of the NADPH oxidase NOX2 in prion pathogenesis. PLoS pathogens *10*, e1004531.

Tamguney, G., Giles, K., Bouzamondo-Bernstein, E., Bosque, P. J., Miller, M. W., Safar, J., DeArmond, S. J., and Prusiner, S. B. (2006). Transmission of elk and deer prions to transgenic mice. Journal of virology *80*, 9104-9114.

Telling, G. C., Scott, M., Hsiao, K. K., Foster, D., Yang, S. L., Torchia, M., Sidle, K. C., Collinge, J., DeArmond, S. J., and Prusiner, S. B. (1994). Transmission of Creutzfeldt-Jakob disease from humans to transgenic mice expressing chimeric human-mouse prion protein. Proceedings of the National Academy of Sciences of the United States of America *91*, 9936-9940.

Telling, G. C., Scott, M., Mastrianni, J., Gabizon, R., Torchia, M., Cohen, F. E., DeArmond, S. J., and Prusiner, S. B. (1995). Prion propagation in mice expressing human and chimeric PrP transgenes implicates the interaction of cellular PrP with another protein. Cell *83*, 79-90.

Thackray, A. M., Klein, M. A., Aguzzi, A., and Bujdoso, R. (2002). Chronic subclinical prion disease induced by low-dose inoculum. Journal of virology *76*, 2510-2517.

Thackray, A. M., Klein, M. A., and Bujdoso, R. (2003). Subclinical prion disease induced by oral inoculation. Journal of virology *77*, 7991-7998.

Wadsworth, J. D., Joiner, S., Linehan, J. M., Desbruslais, M., Fox, K., Cooper, S., Cronier, S., Asante, E. A., Mead, S., Brandner, S.*, et al.* (2008). Kuru prions and sporadic Creutzfeldt-Jakob disease prions have equivalent transmission properties in transgenic and wild-type mice. Proceedings of the National Academy of Sciences of the United States of America *105*, 3885-3890.

White, M. D., Farmer, M., Mirabile, I., Brandner, S., Collinge, J., and Mallucci, G. R. (2008). Single treatment with RNAi against prion protein rescues early neuronal dysfunction and prolongs survival in mice with prion disease. Proceedings of the National Academy of Sciences of the United States of America *105*, 10238-10243.

Zhu, C., Herrmann, U. S., Falsig, J., Abakumova, I., Nuvolone, M., Schwarz, P., Frauenknecht, K., Rushing, E. J., and Aguzzi, A. (2016). A neuroprotective role for microglia in prion diseases. The Journal of experimental medicine *213*, 1047-1059.
